# Innovations in oral hygiene tools: a mini review on recent developments

**DOI:** 10.3389/fdmed.2024.1442887

**Published:** 2024-08-14

**Authors:** Sucharitha Palanisamy

**Affiliations:** Department of Periodontics and Oral Implantology, SRM Dental College and Hospital, Chennai, India

**Keywords:** toothbrush modifications, newer oral hygiene strategies, oral health technology, dental hygiene innovations, smart oral care devices

## Abstract

**Background:**

This review examines advancements in oral hygiene aids and their impact on gingival and periodontal health. As periodontal diseases are widespread, effective hygiene is vital. Enhancements in traditional tools and innovations have improved oral hygiene status. Clinical evidence confirms these innovations improve gingival and periodontal health, though proper use and patient adherence are crucial.

**Methodology:**

A comprehensive literature search was conducted using MeSH terms like “Gingivitis/prevention & control*”, “Dental Plaque/prevention & control*”, “Dentifrices”, “Electrical Equipment and Supplies*”, “Toothbrushing”, “Equipment Design”, “Anti-Infective Agents/therapeutic use”, “Oils, Volatile/therapeutic use”, “Dental Devices, Home Care”, “Dentifrices*/therapeutic use”, “Vibration”, “Gingivitis*/drug therapy”, “Gingivitis*/prevention & control”, “Gingival Hemorrhage/prevention & control”, “Anti-Inflammatory Agents/pharmacology”, “Chlorhexidine/pharmacology”, “Mouthwashes/pharmacology”, “Anti-Bacterial Agents/pharmacology”, “Mouthwashes/therapeutic use”, “Anti-Infective Agents, Local*/therapeutic use”, “Mouthwashes/chemistry*”, “Plant Extracts/therapeutic use*”, “Sodium Dodecyl Sulfate/therapeutic use*”, “Treatment Outcome”, “Oral Hygiene/methods”, “Toothpastes/therapeutic use*”, “Hyaluronic Acid/therapeutic use”, “Chronic Periodontitis*/therapy”, “Periodontal Attachment Loss/therapy”, “Probiotics*/therapeutic use”, “Oral Hygiene*”, “Periodontal Index” and so on. This search utilized PubMed and Google Scholar, restricted to English-language publications from 2018 to 2024. The screening process involved reviewing titles, abstracts, and keywords, focusing on randomized clinical trials only. Inclusion criteria focused on novel innovations in conventional oral hygiene methodologies. A total of 86 randomized clinical trial articles met the inclusion criteria.

**Results:**

Recent innovations in traditional oral hygiene tools have markedly enhanced oral hygiene levels and patient compliance. These newer innovations demonstrate substantial efficacy in plaque control and gingival health. Clinical outcomes underscore their pivotal role in improving oral hygiene standards, promoting reduced gingivitis and enhanced patient adherence to oral care regimens.

**Conclusion:**

Advanced oral hygiene aids significantly improve gingival and periodontal health. However, patient adherence and correct usage are crucial for their optimal performance. Incorporating advanced oral hygiene aids into daily practices is essential for achieving optimal periodontal health, and continuous education is necessary to ensure their effective use.

## 1 Introduction

Periodontal diseases are among the most prevalent chronic health conditions worldwide, significantly affecting individuals’ quality of life and overall health. The primary etiology is the accumulation of bacterial biofilm on tooth surfaces, which initiates inflammation. Inadequate removal of dental deposits and neglect of specific areas can precipitate disease progression, leading to the breakdown of the tooth’s supportive structures and further complications (1). Effective oral hygiene practices are crucial for the prevention and management of periodontal diseases (2, 3). Over time, advancements in oral hygiene aids have markedly improved their effectiveness in maintaining optimal oral health. Traditional methods, including manual toothbrushes, dental floss, and mouth rinses, have long been essential components of daily oral care routines. However, these conventional tools often fail to remove plaque from all areas of the mouth, particularly interdental spaces and subgingival regions (4). Recognizing these limitations, more sophisticated tools have been developed to enhance plaque removal and reduce gingival inflammation. These innovations primarily focus on ergonomic designs that enhance precision, particularly in difficult-to-reach areas (5). Modifications in the shank and head sizes of toothbrushes, along with morphological adaptations in interdental brushes, aim to improve accessibility and cleaning efficacy. Electric toothbrushes featuring oscillating-rotating and sonic technologies have demonstrated superior efficacy in plaque removal compared to manual toothbrushes (6, 7). Interdental brushes and water flossers provide more effective cleaning of interdental spaces than traditional flossing techniques (8). Furthermore, antimicrobial mouthwashes, particularly those containing chlorhexidine, offer significant benefits in reducing periodontal pathogens and controlling gingivitis (9). Clinical research consistently demonstrates that the use of these advanced oral hygiene aids results in improved gingival and periodontal health outcomes, including reduced gingivitis, lower plaque indices, and decreased probing pocket depths. Despite these advancements, their effectiveness depends heavily on patient adherence and proper usage techniques. Therefore, continuous education and training on the correct use of these tools are essential to fully realize their benefits.

**Table 1:** Recent modifications in the oral hygiene aids with references.

Oral hygiene aid	Modifications in oral hygiene aid
Manual Toothbrush	(11, 13, 16, 45, 10, 12, 13, 14, 15, 17, 18, 19, 20, 21, 22, 23)
Powered Toothbrush	(24, 25, 26, 27, 28, 29, 30, 31, 32, 33, 34, 35, 36, 37, 38, 39, 40, 41, 42, 43, 44).
Interdental Cleansing Modalities	(45, 46, 47, 48, 49, 50, 51, 52, 53)
Dental Floss	(54, 55, 56, 57, 58, 59, 60, 61, 62, 63, 64).
Dentifrices, Mouth Rinses and Gels	(65, 66, 67, 68, 69, 70, 71, 72, 73, 74, 75, 76, 77, 78, 79, 80, 81, 82).
Others	(83, 84, 85, 86, 87, 88, 89, 90, 91, 92, 93, 94, 95, 96).

The recent advancements in oral hygiene maintenance tools are detailed below in Table 1.

## 2 Oral hygiene aids

**Figure 1:**
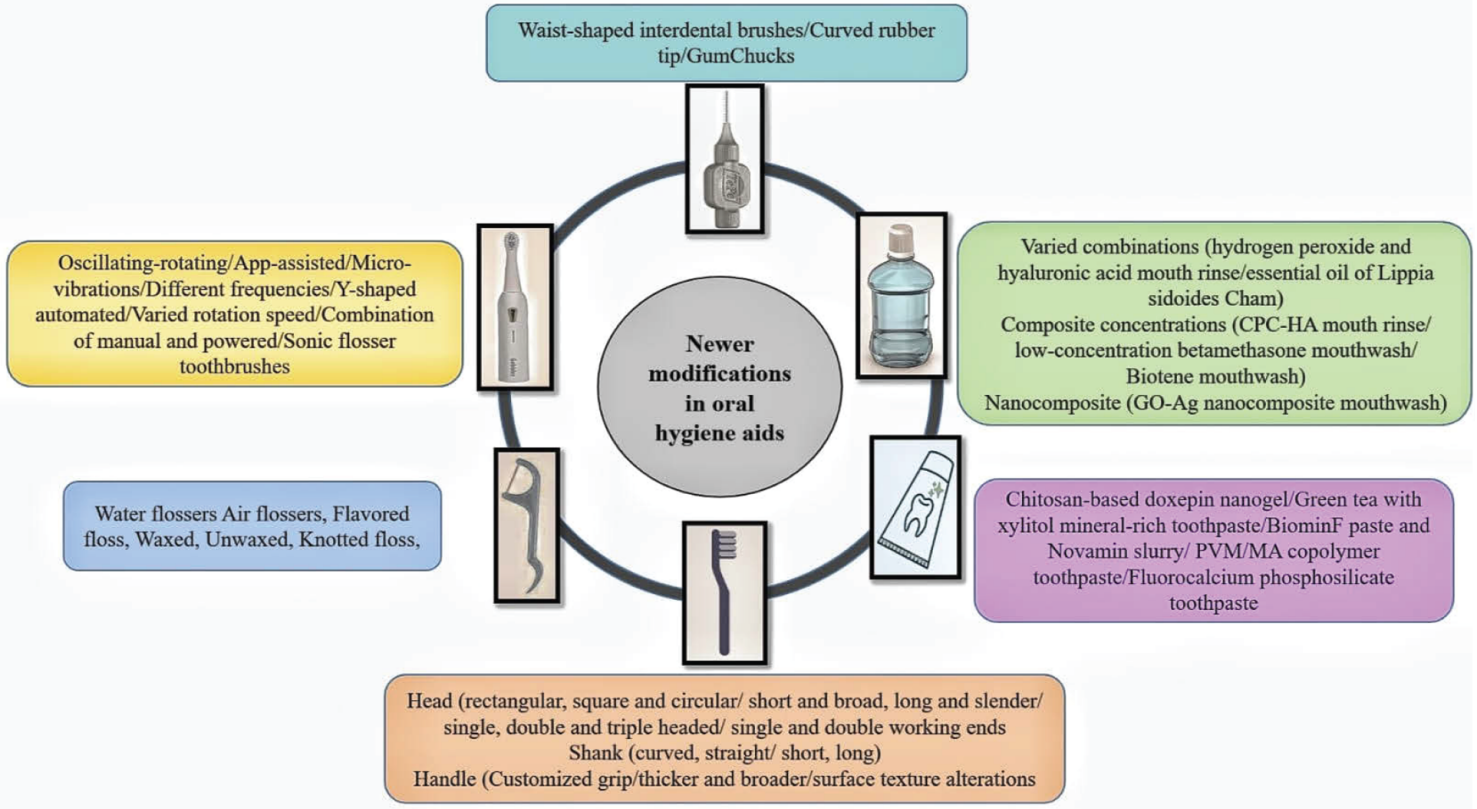
Summary of recent innovations in oral hygiene aids.

Essential to dental care are maintaining optimal oral health and preventing disorders, facilitated by various oral hygiene tools designed for thorough cleaning and plaque removal. These tools range from basic toothbrushes and floss to advanced electric toothbrushes, water flossers, and interdental brushes, each offering unique benefits. Understanding these oral hygiene maintenance modalities is crucial for effective oral care and dental health maintenance (Figure 1).

### 2.1 Manual toothbrushes

The efficacy of plaque removal is influenced by several factors including brushing duration, technique, and pressure, which can vary from person to person. Advancements in toothbrush design, aimed at addressing the limitations of traditional methods, have led to the creation of toothbrushes that are more effective at removing plaque compared to the flat-trimmed versions of the early 20th century. These modern toothbrushes feature a variety of bristle configurations, including different quantities, arrangements, lengths, diameters, and head designs. For instance, a 2018 study by Sandhya P. Naik and colleagues investigated the effectiveness of different bristle designs in individuals with fixed orthodontic appliances. Participants were divided into groups using flat, zigzag, and crisscross bristle toothbrushes. After four weeks, the crisscross bristle toothbrush showed the highest mean plaque reduction (10). Similarly, a 2019 study by Clara S. Kim et al. compared a novel bristleless toothbrush to a soft nylon-bristled one in adults undergoing periodontal maintenance, finding equivalent plaque removal and prevention of gingival inflammation, with potential benefits for gingival tissue recovery (11). Additionally, Zhipeng Xu et al. (2019) demonstrated that a manual toothbrush with CrissCross and tapered bristle technology significantly reduced gingivitis and improved plaque removal compared to a traditional flat-trim toothbrush (12). Research by Sara H. Rosenberg in 2018 showed that smooth handle toothbrushes had significantly lower bacterial contamination compared to grooved handle toothbrushes, based on DNA and endotoxin levels (13). A study by Fathima Fazrina Farook et al. in 2023 found that among patients with fixed orthodontic appliances, the Flat Trim (FT) toothbrush was superior in plaque removal after a single use compared to Cross Action (CA) and Orthodontic Type (OT) toothbrushes (14). Additionally, Vineet Kini et al. (2019) revealed that charcoal-infused bristle toothbrushes were more effective in plaque removal and exhibited less wear over time compared to nylon bristle toothbrushes (15). For children with cerebral palsy, custom-handled toothbrushes significantly improved oral hygiene and reduced bad breath over a week (16). In a similar vein, Dr. Lakshmi Krishnan’s team found that modified manual toothbrushes significantly reduced plaque and gingival scores in adolescents with cerebral palsy (17). Trupthi Rai et al. (2018) concluded that personalized toothbrushes notably enhanced oral hygiene and gingival health in cerebral palsy patients (18). Furthermore, Soncini JA and colleagues (1989) showed that individually modified toothbrushes substantially reduced plaque among cerebral palsy patients during clinic visits and at home (19). Tests of the Balene toothbrush in individuals with acquired brain injuries revealed effectiveness comparable to regular toothbrushes, whether used independently or with assistance (20). Studies also found that nanogold-coated toothbrushes significantly reduced microbial contamination and plaque (21) and that short-headed toothbrushes matched conventional ultrasoft ones in plaque removal, with user preference favoring the former due to its higher bristle count (22). Additionally, research highlighted the superior plaque removal of triple-headed and T-shaped toothbrushes compared to ultra-soft single-headed brushes, with variations in plaque scores noted across different toothbrush types and teeth locations (23).

### 2.2 Powered toothbrushes

Electric toothbrushes, utilizing oscillating-rotating or sonic technologies, outperform manual toothbrushes in plaque removal efficacy. Designed for consistent brushing and reaching difficult areas, they often feature timers and pressure sensors to enhance the user experience. Clinical studies confirm their effectiveness in reducing plaque and gingivitis, thus improving oral health. Gomez-Pereira et al. (2022) evaluated a prototype power toothbrush (PTB) with low rotation speed in “Gumline” and “Interdental” modes, finding that combined modes excelled in plaque removal compared to conventional and commercial PTBs (24). Similarly, Yılmaz Zafer Bilen et al. (2021) studied powered interactive toothbrushes vs. conventional ones during orthodontic treatment, noting improvements in periodontal health, suggesting the viability of powered toothbrushes for maintaining oral health during such treatments (25). An app-assisted interactive powered toothbrush also proved more effective than a manual toothbrush in reducing plaque and gingivitis, as well as preventing gingival abrasion (26). Klonowicz et al. (2018) tested a hybrid toothbrush adaptable for manual, sonic, or combined usage, finding it particularly effective in combined mode for plaque removal after a single use (27). An oscillating-rotating electric toothbrush with a unique round brush head also achieved superior plaque and gingivitis reduction compared to a manual toothbrush (28). Additionally, sonic-flosser toothbrushes with full-size brush heads were significantly better at improving gingival health and reducing plaque compared to manual toothbrushes and dental floss (29). Jing LV et al. (2018) found a novel high-frequency sonic toothbrush more effective at reducing plaque and equally effective at reducing gingivitis over six months compared to an oscillating-rotating toothbrush and a conventional sonic toothbrush (30). For children, a powered toothbrush developed by Mary Francis et al. (2021) showed potential for significant oral hygiene improvements (31). Erden and Camcı (2024) noted superior plaque elimination with an interactive electric toothbrush among orthodontic subjects but found no definitive link between toothbrush type and gingival index or specific salivary bacteria (32). Finally, Ralf Adam et al. (2020) found that the novel O-R toothbrush with micro-vibrations resulted in a significantly greater plaque reduction compared to the manual toothbrush (33). Barallat Lucia et al. (2022) compared an updated sonic toothbrush with a manual one, finding the sonic toothbrush more effective in reducing plaque after a single use (34). In another study, CUdent manual toothbrushes and the GoodAge triple lock toothbrush were equally effective in plaque removal and reducing gingival irritation among the elderly, with CUdent excelling in buccal plaque removal (35). Wang et al. (2022) found a compacted dual-head power toothbrush (DH) superior to a single-head (SH) in plaque removal among university students (36). Milleman et al. (2020) showed the ToothWave radiofrequency (RF) toothbrush significantly reduced plaque, calculus, and gingival inflammation compared to an ADA-approved powered toothbrush (37). Nevins et al. (2021) observed greater improvements in plaque and gingival inflammation with an ionic-sonic electric toothbrush compared to a manual one after one week (38). Takenouchi et al. (2021) highlighted the potential efficacy of high-frequency ultrasound toothbrushes in oral hygiene (39). Koşar et al. (2020) found no significant improvements with xylitol-infused toothbrushes in orthodontic patients (40). Statie et al. (2024) noted a Y-shaped automated electric toothbrush was superior to no brushing but inferior to manual brushing (41). Mylonopoulou et al. (2021) found no significant difference in efficacy between electric 3D and manual toothbrushes in orthodontic patients (42). Adam et al. (2020) and Grender et al. (2020) both demonstrated the superior performance of oscillating-rotating electric toothbrushes with micro-vibrations in reducing plaque and gingivitis compared to manual and sonic toothbrushes (43, 44).

### 2.3 Interdental cleansing modalities

Interdental cleaning methods encompass a range of tools crucial for thorough interdental hygiene. From conventional dental floss to modern water flossers and air flossers, each addresses specific needs. Specialized tools like rubber tip stimulators and oral irrigators offer tailored solutions. The choice depends on personal preference, interdental spaces, and dental needs, underscoring the guidance of dental professionals. Research indicates the efficacy of various methods in reducing gingivitis and plaque. Mirza et al. (2024) found the Philips Sonicare Cordless Power Flosser alongside a manual toothbrush notably reduced gingival inflammation (45). Moretti et al. (2020) showed dental floss and curved rubber bristle interdental cleaners to be equally effective (46). Li et al. (2024) highlighted the efficacy of the Philips Sonicare Power Flosser (47). Wehner et al. (2021) observed comparable plaque control between different interdental brushes (48). Graziani et al. (2018) demonstrated the effectiveness of various regimens in reducing plaque and gingival irritation (49). Pune N Paqué et al. (2020) concluded that waist-shaped interdental brushes cleaned proximal tooth surfaces better than cylindrical ones (50). Lastly, Erbe et al. (2023) and Hennequin-Hoenderdos et al. (2018) emphasized the efficacy of specific interdental brushes in reducing plaque and inflammation, respectively (51, 52). Stauff et al. (2018) provided insights into alternative devices for those struggling with traditional flossing methods (53).

### 2.4 Dental floss

Dental floss, available in various forms such as waxed, unwaxed, and flavored, plays a vital role in removing plaque and food debris between teeth. Waxed floss is ideal for narrow spaces, while unwaxed provides better traction for thorough cleaning. Flavored floss enhances user satisfaction, promoting consistent use. Specialized options, including dental tape and super floss, cater to specific needs like bridgework. Roa López et al. (2021) found knotted floss comparable to conventional floss for plaque removal, especially for beginners (54). Tyler et al. (2023) reported no additional benefits of using a manual toothbrush alongside a WaterPik® for fixed orthodontic appliances (55). Gomes et al. (2022) concluded that knotted flossing is as effective and safe as conventional flossing in reducing plaque and gingival inflammation (56). Muniz et al. (2018) noted that 2% chlorhexidine digluconate-infused floss significantly reduced supragingival interproximal biofilm (57). Studies by Wiesmüller et al. (2023) and Xu et al. (2023) demonstrated the effectiveness of oral irrigators and daily water flossing in managing gingival health (58, 59). Further, Araújo et al. (2020) and Mancinelli-Lyle et al. (2023) highlighted the benefits of text reminders and advanced flossing tools in periodontal health (60, 61). Lin et al. (2020) found GumChucks preferable for children’s plaque removal, and AlMoharib et al. (2024) observed both water and interdental flossing effective during orthodontic treatment (62, 63). Goyal et al. (2018) showed significant improvements in gingival health with combined Waterpik® and manual brushing (64).

### 2.5 Dentifrices, mouth rinses and gels

Dentifrices, mouthwashes, and gels provide various benefits for oral care, addressing needs like caries prevention, sensitivity relief, and bacterial control, with choices often guided by dental professionals. Kaur et al. (2021) found that a novel dental gel reduced probing depths and inflammation in periodontitis patients without initial scaling and root planing (65). Li et al. (2024) demonstrated a toothpaste’s effectiveness and safety for dentinal hypersensitivity (66). Newman et al. (2022) highlighted a mouth rinse that reduced plaque re-accumulation, though potential adverse effects were noted (67). Samiraninezhad et al. (2023) introduced a chitosan-based doxepin nanogel for oral mucositis, and Boccalari et al. (2022) found a hydrogen peroxide and hyaluronic acid mouth rinse effective for gingivitis (68, 69). Tadakamadla et al. (2020) revealed a CPC-HA mouth rinse was as effective as CHX in preventing plaque and gingivitis without staining (70). Soundarajan and Rajasekar (2023) developed a GO-Ag nanocomposite mouthwash for gingivitis, while Saliasi et al. (2018) and Montesani et al. (2024) tested formulations reducing gum bleeding and plaque (71–73). Research has scrutinized diverse toothpaste formulations, evaluating mouthwashes like “green tea” and “green tea with xylitol,” mineral-rich toothpaste for remineralization, and BiominF paste and Novamin slurry for orthodontically-induced white spot lesions. Studies have also examined toothpaste with PVM/MA copolymer for enamel erosion-rehardening, natural ingredient toothpaste for plaque and gingivitis improvement, and fluorocalcium phosphosilicate toothpaste for dentin hypersensitivity and acid erosion (74–79). Additionally, research has investigated the soft tissue desquamation from toothpaste, a post-foaming dental gel for reducing localized gingival inflammation, and the comparison between a new radiofrequency toothbrush and a sonic vibrating toothbrush for tooth stain reduction and shade improvement (80–82).

### 2.6 Others

Various combinations of interdental aids with manual or powered toothbrushing have been tested to improve oral hygiene, integrating automatic cleansing devices to enhance Oral Health-Related Quality of Life (OHRQoL). Educational initiatives promote awareness and patient motivation for optimal oral hygiene. Kimberly R. Milleman’s 2023 research found the Fresh Health Inc. system with manual toothbrushing (Fresh + MTB) superior to string floss with manual toothbrushing (floss + MTB) and manual toothbrushing alone (MTB) in reducing gingivitis, plaque, pocket depth, and bleeding on probing (BOP) (83). Keller et al. (2023) reported that while manual brushing reduced more plaque overall, the Y-brush had similar potential with better fit (84). Noraida Mamat (2022) found a T-shaped toothbrush improved children’s gingival health and plaque clearance (85). Padmini Hari’s 2021 study showed the SUN Teeth™ toothbrush was as effective as a conventional ADA toothbrush (86). Other studies (87–90) explored various innovative toothbrushes and incentives, with mixed results on plaque reduction and brushing frequency. Schnabl et al. (2021) compared a “ten seconds” auto-cleaning device to uninstructed manual toothbrushing, finding manual brushing more effective, highlighting the need for improved bristle design (91). Yang et al. (2024) evaluated a tooth-brushing guidance system in preschool children, revealing enhanced plaque removal in difficult areas like the tongue and palate (92). Sabbagh et al. (2020) studied Salvadora persica (miswak) sticks vs. fluoridated toothpaste, finding both effective in reducing plaque scores, with miswak also beneficial for salivary bacteria associated with lower caries risk (93). Saraf et al. (2023) assessed a cartoon-based educational aid vs. a conventional acrylic brushing model for preschoolers, with both methods effectively reducing plaque scores (94). Vouros et al. (2022) evaluated a protocol combining an air-abrasive device with ultrasonic instrumentation (GBT) vs. traditional Scaling and Root Planing (SRP), showing similar effectiveness but with shorter treatment times and better patient perception (95). Weber et al. (2024) investigated antimicrobial chewing gum’s impact on orthodontic patients, finding both experimental and control gums equally effective in reducing plaque and gingival inflammation, and improving OHRQoL (96).

## 3 Conclusion and future perspectives

The field of oral hygiene is undergoing rapid transformation, propelled by technological advancements, increased recognition of oral health’s integral role in overall well-being, and a growing demand for more effective and user-friendly products. Contemporary modifications in oral hygiene tools, such as electric toothbrushes with smart technology, water flossers, and interdental cleaners, have markedly improved the efficacy of daily oral care routines. These innovations provide more personalized, efficient, and accessible solutions, catering to diverse dental needs and preferences. These newer modifications play a pivotal role in the orthodontic-perio and prosthodontic-perio relationship, significantly enhancing plaque control and reducing inflammation, which are essential for maintaining oral health. In orthodontics, advanced tools like electric toothbrushes, interdental brushes, and water flossers enable patients with braces to clean hard-to-reach areas effectively, preventing gingivitis and periodontitis. In prosthodontics, antimicrobial mouthwashes and specialized cleaning devices promote the longevity and health of dental prostheses by minimizing bacterial accumulation around implants and prosthetic margins (97, 98). These innovations support the intricate balance between orthodontic and periodontal health, as well as prosthodontic and periodontal health, by fostering healthier gums and reducing the risk of periodontal complications. The incorporation of artificial intelligence and machine learning in oral hygiene devices is particularly promising, offering real-time feedback and tailored recommendations that enhance oral health outcomes. Furthermore, the development of eco-friendly and sustainable oral care products highlights a burgeoning commitment to environmental stewardship. Looking ahead, future advancements are anticipated to further refine these technologies, enhancing user experience, improving accessibility for underserved populations, and mitigating environmental impact. The integration of biotechnology, including probiotics and advanced biomaterials, holds substantial potential to revolutionize preventive care and treatment options, promoting a holistic approach to oral health. Sustained research and interdisciplinary collaboration will be essential in driving these innovations, ensuring that oral hygiene aids not only fulfill the evolving needs of consumers but also contribute to broader health and sustainability objectives. As oral health continues to be increasingly recognized as vital to overall health, the ongoing evolution of oral hygiene aids is poised to play a crucial role in fostering a healthier, more informed, and environmentally conscious society.
