# Corrigendum: Innovations in oral hygiene tools: a mini review on recent developments

**DOI:** 10.3389/fdmed.2025.1577857

**Published:** 2025-03-12

**Authors:** Sucharitha Palanisamy

**Affiliations:** Department of Periodontics and Oral Implantology, SRM Dental College and Hospital, Chennai, India

**Keywords:** toothbrush modifications, newer oral hygiene strategies, oral health technology, dental hygiene innovations, smart oral care devices

A Corrigendum on Innovations in oral hygiene tools: a mini review on recent developments By Palanisamy S (2024). Front. Dent. Med. **5**:1442887. doi: 10.3389/fdmed.2024.1442887

In the published article, there was an error reporting the details in reference 33. A correction has been made in paragraph **2 Oral hygiene aids**, *2.2 Powered toothbrushes*. This sentence previously stated:

“Finally, Ralf Adam et al. (2020) found that a manual toothbrush recommended by the ADA was superior in plaque reduction compared to an innovative oscillating-rotating powered rechargeable toothbrush with micro-vibrations (33).”

The corrected sentence appears below:

“Finally, Ralf Adam et al. (2020) found that the novel O-R toothbrush with micro-vibrations resulted in a significantly greater plaque reduction compared to the manual toothbrush (33).”

The authors apologize for this error and state that this does not change the scientific conclusions of the article in any way. The original article has been updated.

